# A study of psychological pain in substance use disorder and its relationship to treatment outcome

**DOI:** 10.1371/journal.pone.0216266

**Published:** 2019-11-07

**Authors:** Steven Mee, Blynn G. Bunney, Ken Fujimoto, John Penner, Garrett Seward, Keeley Crowfoot, William E. Bunney, Christopher Reist

**Affiliations:** 1 Applied Innovative Psychiatry, Los Alamitos, California, United States of America; 2 Department of Psychiatry, University of California Irvine, Irvine, California, United States of America; 3 Loyola University, Chicago, IIlinois, United States of America; The University of Chicago, UNITED STATES

## Abstract

Substance Use Disorder (SUD) is a major public health concern affecting an estimated 22.5 million individuals in the United States. The primary aim of this study was to characterize psychological pain in a cohort of patients participating in outpatient treatment for SUD. A secondary aim was to determine the relationships between pre-treatment assessments of psychological pain, depression, anxiety and hopelessness with treatment retention time and completion rates. Data was analyzed from 289 patients enrolled in an outpatient community drug treatment clinic in Southern California, U.S. A previously determined threshold score on the Mee-Bunney Psychological Pain Assessment Scale (MBP) was utilized to group patients into high and low-moderate scoring subgroups. The higher pain group scored higher on measures of anxiety, hopelessness and depression compared to those in the low-moderate pain group. Additionally, patients scoring in the higher psychological pain group exhibited reduced retention times in treatment and more than two-fold increased odds of dropout relative to patients with lower pre-treatment levels of psychological pain. Among all assessments, the correlation between psychological pain and treatment retention time was strongest. To our knowledge, this is the first study to demonstrate that psychological pain is an important construct which correlates with relevant clinical outcomes in SUD. Furthermore, pre-treatment screening for psychological pain may help target higher-risk patients for clinical interventions aimed at improving treatment retention and completion rates.

## Introduction

Substance Use Disorder (SUD) is a major public health concern affecting an estimated 22.5 million individuals in the United States [1]. In 2017, 70,237 deaths were attributed to drug overdoses—a significant increase of 9.6% over the past year [2]. Nationally, substance-related addiction incurs a financial burden exceeding $400 billion per year including expenses related to lost work productivity, healthcare and drug-related crime [3]. There are 14,500 drug treatment centers in the US but only a relatively small number of individuals (11%) enter treatment despite the fact that many programs are supported by local, State and Federal government funding [4]. Drug treatment programs continue to strive for improving program outcomes, however, data shows that they are maximally effective when patients remain in treatment (length of stay-LOS) for an average of 90 days or more [5, 6]. Program completion is associated with better health, fewer readmissions, less criminal activity [7] and lower mortality rates [8]. Poor retention rates (inadequate LOS) and failure to complete (Dropout) pose major clinical challenges [9] to successful substance treatment. Of those SUD individuals entering a program, 17–67% complete treatment depending on the substance of choice and whether therapy is offered as in- or outpatient [7]. Efforts to identify factors predictive of treatment non-completion have shown that demographics alone are relatively poor indicators while inadequate length of stay (LOS) negatively impacts treatment outcomes. [7, 10].

Psychological pain has emerged as an important clinical psychometric construct. Attempts to measure and characterize psychological pain began with early investigations into its impact on suicidality and more recently in populations suffering from Major Depression [11, 12],[13–21]. It has been characterized by investigators as an ‘Emotionally-based, extremely aversive feeling’ and ‘A response to noxious psychological stimuli, analogous to physical pain that…may operate on a continuum from mild to severe.’ Other groups have defined psychological pain as ‘The hurt, anguish, soreness, aching, psychological pain in the psyche.’ Still others as ‘A lasting, unpleasant and unsustainable feeling characterized by a perception of inability or deficiency of the self…’ [22–24]. Investigators have variously referred to this form of pain as mental pain, emotional pain, social pain or psychache. The construct models reveal two broad categories of conceptual framework: 1) a more generalized negative internal response to real or perceived deficiencies of self and 2) a narrower, negative emotionally-based aversive response to internal and external stressors most analogous to physical nociception. The disparate state of construct understanding and lack of cohesive definition reveals a field that is maturing but requires more research to develop a truly unified definition and discern its relationship to other broader theories of negative emotional responsiveness such as Negative Affectivity (37) and internalizing psychopathology. We propose that the narrower concept of psychological pain as an aversive signal analogous to physical pain and similarly generative of profoundly negative emotional experiences, allows for consideration of its relationship to suicidality and possibly SUD as a means to escape, moderate or otherwise control psychological pain. Further data are required to mature the construct and elucidate its relationship to related concepts of NA and internalizing psychopathology.

There is very little data characterizing psychological pain in SUD. Guimaraes et al. assessed psychological pain in a population undergoing treatment for SUD using a translated (English to Portuguese) abbreviated version (24 items) of the Orbach & Mikulincer Mental Pain Scale (OMMP), finding a small to moderate positive correlation between mental pain and the severity of addiction [25]. A Norwegian study addressing ‘mental distress’ and variables related to dropout included 454 patients from five inpatient SUD treatment centers using a brief version (10-item) of the Hopkins Symptom Checklist (HSCL). The HSCL assesses obsessive-compulsivity, somatization, anxiety and depression. A high score on the HSCL, which the investigators interpreted as ‘mental distress’, was associated with treatment dropout [26]. The HSCL, however, does not specifically define or claim to assess psychological pain. Some have speculated that use of addictive substances to suppress negative emotions so that the control of mental pain (e.g., drug seeking induced by acute stress) may be the objective of substance use behaviors, rather than the pleasure-seeking associated with substance use [27]. Conclusive evidence characterizing psychological pain in SUD and documenting its clinical implications is largely lacking. In its absence, a convergence of evidence supporting a link through shared clinical comorbidities of depression and suicidality as well as data from at least one study, encourage efforts to further our understanding. Previously, we documented that patients experiencing a Major Depressive Episode reported elevated psychological pain relative to healthy controls. Psychological pain was significantly correlated with both the intensity of depressive symptoms as well as suicidal ideation(10). Further, 87% of subjects reporting the highest category of psychological pain (MBP ≥ 32) also met DSM-IV criteria for a current Major Depressive Episode. A follow-up study performed in a separate population of acutely suicidal U.S. Veterans, found psychological pain to be correlated with depression and suicidality scores (11). Moreover, those patients with future suicidal behavior observed over an 18-month observation period all had high scores of psychological pain. These findings are consistent with previous reports demonstrating a correlation between psychological pain, depression and suicidality (11–13), albeit with fewer efforts focused on specific mood disorder populations (17). Depression and elevated suicidality, both shown to be correlated with psychological pain, are also commonly seen in patients with SUD (35,36). The phenomenological and clinical overlap between depression, suicidality, psychological pain and SUD suggests that psychological pain is elevated in SUD and that higher levels of pain could impede treatment. Although comprehensive characterizations of psychological pain and potential impacts on SUD treatment are lacking, a growing convergence of circumstantial, clinical and theoretical evidence supports further investigation of these questions and is the impetus for the current study.

This study was undertaken to characterize psychological pain in a population undergoing treatment for SUD. We hypothesized that psychological pain scores would be correlated with assessments of depression and anxiety as we observed in previously studied depressed and suicidal clinical populations [11, 12]. The MBP is a brief 10-item self-report instrument developed for use in a variety of clinical settings. It defines the construct of psychological pain for patients to use when answering the items as ‘Intense psychological pain is a feeling which is experienced as unbearable torment. It can be experienced during a psychiatric disorder or a tragic loss such as the death of a child… circle the number that best describes how often you experience severe psychological pain.’ Items are rated on a 5-point Likert Scale and include content examining current and past (within 3 months) psychological pain in terms of frequency, intensity levels, and perceived tolerance (e.g., how much psychological pain can you tolerate before it becomes unbearable?). Administering the scale to a population diagnosed with current Major Depressive Episodes (MDE), we found increased levels of psychological pain in depressed patients compared with healthy controls [12]. Secondary findings included significant correlations between psychological pain, depression, hopelessness and suicidality scores obtained from the Suicide Behavior Questionnaire [28]. In a follow-up study examining psychological pain as a pre-treatment risk indicator for suicidality and serious suicide attempts in U.S. Veterans admitted to a suicide prevention program, findings showed that psychological pain accounted for more shared variance with suicidality than assessments of depression, hopelessness and impulsivity. In addition, we identified a subgroup of suicidal patients scoring highest in psychological pain (24/57) by applying a previously tested screening score of MBP≥32 [defined as 0.5 SD above the mean of MDE patients [12]], which were predicted to be at highest risk for negative treatment outcomes. At a 15-month follow-up, 9 of these 24 higher scoring patients experienced a documented serious suicidal event (as defined by criteria on the C-SSRS) and 7/9 would have died if not found (One patient completed suicide). Taken together, these results provided preliminary evidence that stratifying patients using psychological pain scores could inform risk determination efforts in identifying patients at higher risk for negative clinical outcomes and concentrated comorbid symptom acuity.

In this study, we evaluated pretreatment assessments of psychological pain, depression, anxiety and hopelessness in a substance addicted outpatient treatment population. We hypothesized that psychological pain would correlate with ratings of co-administered symptom assessments as we observed in previous findings from depressed, suicidal and healthy control populations. In addition, we tested whether a subgroup of highest scoring SUD patients in terms of pre-treatment psychological pain would be associated with greater severity of co-assessed symptoms and elevated risk for poorer treatment outcomes (treatment retention times and completion rates) relative to lower scoring patients.

## Methods

### Study design

A retrospective analysis of medical records was conducted for patients enrolled between 2011–2013 in the Substance Abuse Counseling Systems of The Gary Center (La Habra, California (SACS); a community-based outpatient SUD treatment program. Data collected included demographics, standardized clinical assessments and outcome variables including completion/dropout status and length of stay (LOS). Patients were referred to the SACS program by medical providers, regional non-profit centers, Orange County (OC) courts, legal agencies and the OC Healthcare Agency. In addition, the SACS treatment program was advertised on the internet (http://orange.networkofcare.org/mh/services/agency.aspx?pid=TheGaryCenterSACS_348_2_0). The Institutional Review Board (IRB) of the County of Orange Healthcare Agency approved the study and waived informed consent due to the minimal risk associated with a retrospective chart review. We carefully protected the identity of the patients by assigning each patient chart record a numerical code to ensure privacy. Research personnel conducting chart reviews were blind to the study protocol.

### Subjects

Medical records (N = 529) from January 2011 to December 2013 for male and female patients ≥18 years of age and meeting the DSM-IV criteria for Substance Dependence or Substance Abuse were screened for inclusion in the study. Patients with incomplete medical records or who did not meet admission requirements were excluded from the study so that a total of 289 patient clinical charts were entered into the analyses. Successful program completion was defined as fulfilling all required elements of the clinical protocol. Data collected in the retrospective chart review included demographics, program length of stay (LOS), completion status and data from clinical rating scales. Detailed socioeconomic variables such as employment, education and marital status were not available. All patients entering the program underwent drug screening at admission and during the course of treatment for alcohol, tetrahydrocannabinol (THC), methamphetamine, cocaine, opiates and benzodiazepines.

#### Exclusion criteria

Subjects under age 18 and those who had not agreed to each required random drug screening as well as clinical assessment were excluded from the retrospective analyses as were those chart records with missing assessment and/or data relevant to completion status.

### Assessment

Data collected from the intake assessment upon admission included ratings from the Mee-Bunney Psychological Pain Assessment Scale (MBP) [11, 12] the Beck Depression Inventory (BDI)[29], the Beck Hopelessness Scale [30] and the Beck Anxiety Inventory (BAI) [31]. The MBP is a ten-item instrument developed to rapidly assess current and recent psychological pain in general clinical populations. Broadly, scale items query the intensity of current and recent pain, ask the respondent to separately consider psychological pain from any co-experienced physical pain and address perceived tolerance to current or future psychological pain. Examples of each category of item content include: “Circle the number that describes your psychological pain at its worst intensity in the last 3 months”, “Compared to the worst physical pain you can imagine, how would you rate your psychological pain at the present time?”, and “How much more psychological pain do you think you can tolerate before it becomes unbearable?” Random drug testing conformed to the standards of the Department of Transportation (DOT)-regulated biological fluid testing and included both observed urine and saliva collection.

#### Program completion

Completion was task dependent and determined by successfully completing the core programmatic components as designed by the SACS treatment team. Primary required elements included: attendance in the program >90 days, participation in 24 group sessions (16 process groups and 8 relapse prevention groups); four individual psychotherapy sessions, evidence of weekly attendance at community-based 12-step programs; two psycho-educational classes; and six random, observed drug tests. In order to maximize the opportunity to complete the treatment program and to accommodate relatively brief diversions from treatment (i.e., court hearings and child visitation), there was no predetermined maximum time for completion.

#### Program dropout

Non-completion status was defined as not completing the tasks necessary for program completion (described above) and/or non-attendance for greater than 30 days.

### Statistical methods

Various statistical analyses were performed with IBM SPSS software. The type I error rate was set at .05 for all analyses. Chi-square analyses were conducted to study the association between whether patients withdrew from treatment (i.e., dropout) and gender, and between dropout and whether patients experienced high or low-to-moderate psychological pain (i.e., patients dichotomized with respect to psychological pain). Between-group *t-*tests were conducted to compare the differences between two groups with respect to continuous variables, such as testing for a gender difference in Length of Stay (LOS). Pearson correlations were calculated to determine the linear relationship between two continuous variables. A logistic regression was conducted to examine the effect of psychological pain (high vs low-to-moderate) on dropout. A Kaplan-Meier Survival (Retention) analysis was conducted to examine the effect of psychological pain (high vs low-to-moderate) on LOS, with the null hypothesis assuming that psychological pain had no impact on LOS.

## Results

### Demographics

Data from 289 patients (212 males and 77 females) were included in the analyses (Table 1 and S1 File). Patients self-identifying as Hispanic comprised a slight majority of the population (55%). Methamphetamine was the most frequently reported substance used (Polysubstance and as drug of choice) (82.4%) followed by alcohol (64.7%) and cannabis (56.4%). The majority of patients were polysubstance users (n = 228; 78.9%), while 21.1% (n = 61) reported using a single drug of choice. The combined number of drugs used by patients ranged from two (n = 116; 40.1%) to five (n = 10; 3.5%) with the majority using two substances, methamphetamine and alcohol.

**Table 1:** Demographic and substance use characteristics.

**Gender /mean age ± SD**	**N**	**Percent (%)**
Males (32.8yrs± 10.9)	212	73.4
Females (34.7yrs ± 10.1)	77	26.6
Combined (33.5yrs ±10.7)	289	100.0
**Ethnicity**	**N**	**Percent (%)**
Caucasian	100	34.6
Hispanic	159	55.0
Black	12	4.2
Asian	7	2.4
Pacific Islander	2	0.7
Other	9	3.1
**Drug of choice with (+) or without (-) polysubstance**		
Alcohol	205	70.9
(+)	187	91.2
(-)	18	.09
Tetrahydrocannabinol (THC)	178	61.6
(+)	163	91.6
(-)	15	8.4
Methamphetamine	238	82.4
(+)	213	89.4
(-)	25	11
Cocaine	68	23.5
(+)	66	97.1
(-)	2	2.9
Opiates	51	17.6
(+)	0	17.6
(-)	51	0
Benzodiazepines	8	2.8
(+)	7	87.5
**-** drug	1	12.5
**Number of drugs used**		
One	61	21.1
Two	116	40.1
Three	64	22.1
Four	38	13.2
Five	10	3.5

### Clinical ratings

As described in Table 2 scoring of clinical assessments for all patients indicated low levels of depression (BDI), anxiety (BAI) and hopelessness (BHS). Psychological pain scores were in the low-moderate range based on previous studies in normal and depressed populations (Mee, et al., 2011). The estimates of Cronbach’s alpha indicated high internal reliability for all assessment instruments: MBP = .902, BDI = .941, BHS = .876, BAI = .958.

**Table 2:** Mean scores for Mee-Bunney Psychological Pain Assessment Scale (MBP), Beck Depression Inventory (BDI), Beck Anxiety Inventory (BAI) and Beck Hopelessness Scale (BHS).

Scale	Score (±SD)	Scoring Category
MBP	23.57 (8.87)	Low-moderate
BAI	11.98 (14.46)	Mild
BHS	4.8 (4.48)	Mild
BDI	8.54 (6.60)	Minimal

The correlations among the clinical assessments are described in Table 3. The strongest relationship was between psychological pain (MBP) and depression (BDI), and the relationship was positive.

**Table 3:** Correlations among the Mee-Bunney Psychological Pain Assessment Scale (MBP), Beck Depression Inventory (BDI), Beck Hopelessness Scale (BHS), Beck Anxiety Inventory (BAI) and treatment program Length of Stay (LOS).

	LOS	MBP	BDI	BHS	BAI
LOS	1.00				
MBP	−.20***	1.00			
BDI	−.17**	.77***	1.00		
BHS	−.11	.63***	.64***	1.00	
BAI	−.14*	.61***	.62***	.44***	1.00

* *p* < .05

**p < .01

***p ≤ .001

#### Completion of treatment and Length of Stay (LOS)

Program completion was defined as satisfying all clinical requirements of the SACS program. Dropouts participated in the program but failed to complete.

#### Completion rates

68 patients (23.5%) completed the treatment program, whereas 221 (76.5%) did not. Significantly fewer patients with higher MBP scores completed the treatment (11.3%) than patients with lower scores did (26.3%). The association between dropout rate and gender was statistically significant, with the dropout rate being higher for males (82.1%) than for females (61.0%), *Χ*^2^ = 12.75, *df* = 1, *p* < .001.

#### Treatment retention/Length of Stay (LOS)

The mean number of days in treatment for all patients (completers and dropouts) was 117.1 days (*SD* = _79.4; range from 7 to 397 days; median number of days spent in treatment was 98 days).

#### Gender

The difference in LOS between female (*M* = 127.9 days, *SD* = 83.4) and male (*M* = 111.2 days, *SD* = 76.7 days) patients was not statistically significant, *t*(287) = 1.71, *p =* .09.

#### Completion status

Completers stayed in treatment for an average of 197.4 days compared to 92.3 days for dropouts, and the difference was statistically significant, *t*(287) = 11.52, *p* < .001. The earliest a patient completed the treatment was in 94 days, whereas the longest a patient required was more than a year (397 days).

#### Psychological pain (MBP) ratings: Completers vs Dropouts

Although overall MBP scores were in the low moderate range for the total patient population (Table 2), patients who dropped out (*M* = 24.4, *SD* = 9.09) were statistically significantly higher on psychological pain than completers were (*M =* 20.9, *SD* = 7.59), *t*(287) = -2.82, *p* = .005. MBP scores were also significantly higher for dropouts with briefer length of stays (LOS < 65 days) than for dropouts with longer LOS (mean MBP _<65LOS_ = 25.9; mean MBP _>65 LOS_ = 22.33; *t*(219) = 3.34, p < .001).

#### Correlation between length of stay (LOS) and ratings for psychological pain (MBP) depression (BDI), anxiety (BAI) and hopelessness (BHS)

LOS was negatively correlated with psychological pain (MBP), depression (BDI), and anxiety (BAI), with the correlations being statistically significant (see Table 3 for the Pearson correlations). LOS was most notably negatively correlated with MBP (*r* = –.20, *p* = .001), then with BDI (*r = –*.17, *p* = .005) and with BAI (*r* = –.14, *p* = .022). LOS was not correlated with hopelessness (BHS; *r* = -.107, *p* = .07).

#### Severity category of psychological pain (MBP) and clinical outcomes

Fifty-three patients (18.3%) met the criterion for high psychological pain—the threshold to consider patients as experiencing high psychological pain (MBP≥32) was established in 2011 and shown to be applicable in two separate clinical populations of 72 and 57 patients (9,10). As illustrated in Table 4, patients experiencing high levels of psychological pain (MBP) at intake also scored statistically significantly higher in depression (BDI), anxiety (BAI) and hopelessness (BHS) compared to patients scoring low-moderate on psychological pain assessment.

**Table 4:** Significant differences in clinical assessment symptom severity between subgroups of patients scoring above and below threshold for high psychological pain (MBP).

Rating Scale	MBP <32 (Low-Moderate) n = 236	MBP ≥32 (High) n = 53	t-test	p-value
BDI	6.44 (Minimal)	17.77 (Mild)	df 278, t = -14.99	p < .001
BAI	8.24 (Minimal)	28.75 (Moderate)	df 278, t = -8.33	p < .001
BHS	3.53 (Minimal)	10.31 (Moderate)	df 253, t = -11.73	p < .001

Table 5 illustrates significant differences in clinical outcomes between patients scoring above and below-threshold for high psychological pain (MBP)

**Table 5:** Significantly fewer high scoring patients on pretreatment psychological pain assessment (MBP ≥ 32) completed treatment than lower scoring patients.

Outcome measure	MBP ≥32 (high) N = 53	MBP <32 (lower) N = 236	p-value
Completers N = 68	6	62	p < .02, df 1, χ^2^ = 5.38
Dropouts N = 221	47	174	p = .02, df 1, χ^2^ = 5.38

#### Completion rates and LOS

A logistic regression analysis was conducted to determine the effect of the dichotomized pre-treatment MBP scores (i.e., high vs. low) on treatment dropout. The results showed that patients scoring high on MBP pre-treatment were more likely to dropout of treatment than patients scoring low on MBP pre-treatment were to, χ^2^ (2) = 5.02, *p* = .025, odds ratio 2.79, relative risk = 1.21. Regarding treatment retention times, patients scoring higher on psychological pain pre-treatment (*M* = 90 days) had a lower LOS than patients scoring lower on psychological pain did (*M* = 123 days), and the trend was statistically significant, *t* (287) = 2.75, *p* = .006. In addition, for only the patients who dropped out of treatment, LOS was statistically significantly shorter for those with high psychological pain (*M* = 73.1 days) than for patients with lower psychological pain (*M* = 97.5 days), *t(*219) = 2.31, *p* = .02. Although patients high in psychological pain exhibited diminished LOS and lower subsequent completion rates, robust increases in both of these variables for patients who remained in treatment for more than 100 days were observed. Specifically, when LOS was greater than 100 days, completion rates for the patients high in psychological pain increased from 11.3% to 35.3% and the low-moderate pain group increased from 26.3% to 48.3%. Overall, 96.8% of all completions for both groups occurred after 100 days of treatment.

#### Retention curves and survival analyses

**Fig 1:**
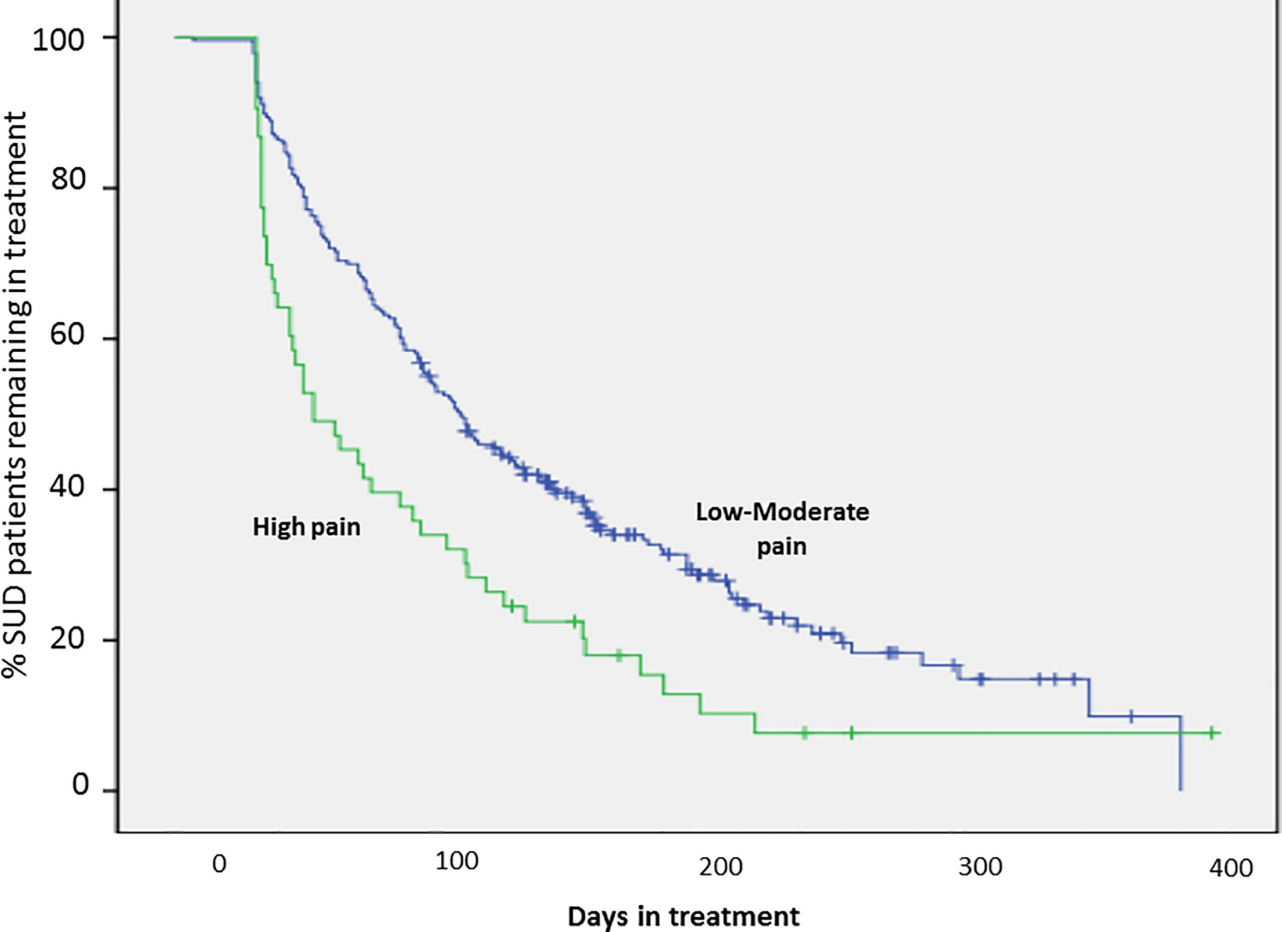
Treatment retention (Kaplan-Meier survival) curves illustrated for SUD patients categorized as High or Low-moderate by psychological pain assessment at program admission.

Kaplan-Meier survival analyses and retention curves were estimated to further compare the high (n = 53) and low-to-moderate (n = 236) psychological pain subgroups and to provide a visualization of LOS and dropout patterns. These analyses revealed significant differences between the patient curves (Log Rank p = 0.001) in terms of retention rates and patterns (Fig 1). Even at similar time points, over-representation of completions clustering on the Low-moderate pain curve while largely absent on the High pain curve is visually apparent. A separate analysis performed on the dropout group alone comparing the high and low-to-moderate psychological pain groups reflected a similar difference between the curves (*p* = .011). Sixty-six percent (n = 35) of high scoring MBP patients had dropped out before the first patient completed the treatment program (day 94), and by day 129, 75% of high-category psychological pain patients had dropped out. Patients high on psychological pain reached 50% attrition after just 53 days compared to 108 days for patients lower on psychological pain.

Program retention curves for High and Low-moderate pain categories. Log Rank analysis showed that the curves significantly differed between the high pain and low-moderate pain patient groups (*χ*2 = 11.1, *p* = .001). (+) indicates individual patient-program completion event. There is a notable clustering of patient completions on the low-moderate pain curve while relatively absent on the high pain curve at similar time points.

## Discussion

This study is, to our knowledge, the first to specifically focus on characterizing psychological pain in a population seeking treatment for substance use disorders. Primarily, the data from this effort add to a growing body of evidence that psychological pain is a quantifiable construct in patients suffering from SUD. This study provides preliminary evidence of higher pre-treatment psychological pain measured at intake within eventual program dropouts compared to patients who completed treatment. We believe this modest difference in mean psychological pain scores is primarily of research value, which, if replicated in larger populations supports a role for psychological pain in SUD. Additionally, we found evidence that elevated pretreatment psychological pain is associated with negative treatment outcomes such as diminished treatment retention time (LOS) and reduced likelihood for program completion. We chose program completion as a proximal indicator of overall treatment outcome as follow-up data for maintain abstinence were not available.

Dropout was the most frequent treatment outcome for the SACS patients. This observation agrees with data from the Treatment Episode Data Set (TEDS) published by SAMHSA [32]. Our patient population exhibited dropout rates somewhat higher than some programs reported to the TEDS nationwide, however, dropout rates greater than 50% have been reported from long term outpatient community-based treatment programs (Levin et al, 27). We did find that completion rates in the SACS population nearly doubled when patients remained in treatment at least 100 days, reaching levels in agreement with data from the TEDS Government analysis.

Pre-treatment levels of anxiety, depression and hopelessness for the total treatment population indicated minimal comorbid psychopathology. A simple binary risk stratification method based on psychological pain (MBP scores), previously developed and applied to depressed and suicidal psychiatric populations [11, 12] re-grouped patient data into high and low-moderate categories of pain. The ‘high’ pain category group, relative to the lower pain group, exhibited significantly greater dropout rates, had more severe psychopathology (depression and anxiety) scores as well as a pronounced reduction in LOS. Survival curve analyses confirmed differences in completion patterns and LOS which suggest that our high and low-moderate pain risk categorization scoring method separated patients into two sub-populations differing in treatment outcomes. Incorporating systematic psychological pain screening within current standard intake assessment paradigms, may aid in identifying patients at program entry posing elevated risk for early dropout and offer the potential for outcome modifying interventions such as increasing retention time. Each of the relatively few high-pain category patients who successfully completed the treatment protocol (only 2.1% of the total patient sample) were associated with LOS >129 days; nearly twice the mean LOS for the total high pain population. In contrast, 89% of high pain patients who dropped out of treatment, did so before the first 102 days of treatment.

There are a number of limitations to this modestly sized, retrospective observational study. Caution is warranted in generalizing our results pending replication in larger populations. Future studies would benefit from a prospective design, however, retrospective designs can be appropriately used in the context of multiple outcome measures [33]. Chart data from patients who were non-compliant with program-administered random drug testing or who did not complete the clinical assessments were excluded from the final analyses. This could have introduced bias to the sample in terms of motivation to participate in treatment, maintain sobriety or otherwise impact outcomes measures. Important psychosocial demographic and socioeconomic factors, including employment, marital and educational status or assessment data on addiction severity were not available for inclusion in data analysis due to limitations on what was collected by the SACS clinical treatment protocol. A similar impact on study implications resulted from the limited detail available on substance use history patterns imposed by the naturalistic post-clinical study design. An additional limitation involves the determination of the threshold for separating the highest scoring patients from lower scoring patients in terms of psychological pain (MBP ≥ 32) to inform risk for negative clinical outcomes a priori. This scoring threshold derived from the initial validation study of the MBP (10) where a determination was made by clinical observation and inspection of co-administered scale rating patterns. The threshold was found to be informative in a follow-up study (11) and those collective results informed the decision to apply it to the present population. A goal of this study was to examine the clinical usefulness of our previously tested threshold score of MBP ≥ 32 as a pre-treatment indicator for elevated risk of poorer clinical outcomes (and more intense comorbid psychopathology) relative to lower scoring patients. While these findings support the informativeness of that threshold in a third clinical population, they are currently of primarily clinical interest and value. Absent a complete item validity analysis in a larger population sample, including a confirmatory factor analysis, the validity of the MBP for assessing psychological pain in SUD and the nature of the role of psychological pain in SUD awaits further clarification. An additional limitation involves the likely collinearity between variables included in the regression analyses. Only one predictor could be included in our logistic regression model when the factors that influenced patients dropping out of treatment were investigated. This was because the other predictors (e.g., depression) were moderately correlated with psychological pain (MBP) and thus, the sample size of the study could not offset the correlations between the predictors. Understanding the relationship between psychological pain and treatment dropout is at an early stage and many of the analyses were conducted without controlling for various predictors. A future study with larger samples could incorporate what has been learned to control for other factors. A prior study, however, suggested that the MBP explains additional variance to that contributed by hopelessness and depression (10).

## Conclusion

In this study, we present evidence that elevated pre-treatment psychological pain may negatively impact program completion and LOS in outpatient substance treatment. The highest scoring patients on psychological pain assessment were ultimately 1.21 times more likely to drop out and to participate in treatment significantly fewer days compared with lower pain scoring peers. Whether this reduction in completion rates is a direct or indirect consequence of decreased LOS remains unanswered and further work in larger populations is needed to better understand these relationships. The survival curve analysis demonstrated a preferential clustering of completions on the lower pain group curve and relative lack of completions on the higher pain curve at identical time points. This suggests that a factor involving high psychological pain, apart from reduced LOS, may also negatively influence completion likelihood. Regardless, the study of psychological pain represents a novel area to further our understanding of the unpredictable outcomes in substance use disorders treatment. The subset of patients experiencing very high levels of psychological pain at treatment initiation may be inherently poorer candidates for outpatient substance treatment and early identification could allow for prompt referral to accessing higher levels of care. Efforts to further our understanding on the negative influence of high pre-treatment levels of psychological pain on completion rates and LOS offer additional opportunities for improving substance treatment outcomes.

## Supporting information

S1 File Main data set for use 7-26-19. (XLSX)
